# Mesenchymal Stromal Cells: From Discovery to Manufacturing and Commercialization

**DOI:** 10.1155/2018/4083921

**Published:** 2018-04-11

**Authors:** Amanda Mizukami, Kamilla Swiech

**Affiliations:** ^1^Center for Cell-Based Therapy CTC, Regional Blood Center of Ribeirão Preto, University of Sao Paulo, 14051-140 Ribeirão Preto, SP, Brazil; ^2^Department of Pharmaceutical Sciences, School of Pharmaceutical Sciences of Ribeirao Preto, University of Sao Paulo, 14040-903 Ribeirao Preto, SP, Brazil

## Abstract

Over the last decades, mesenchymal stromal cells (MSC) have been the focus of intense research by academia and industry due to their unique features. MSC can be easily isolated and expanded through *in vitro* culture by taking full advantage of their self-renewing capacity. In addition, MSC exert immunomodulatory effects and can be differentiated into various lineages, which makes them highly attractive for clinical applications in cell-based therapies. In this review, we attempt to provide a brief historical overview of MSC discovery, characterization, and the first clinical studies conducted. The current MSC manufacturing platforms are reviewed with special attention regarding the use of bioreactors for the production of GMP-compliant clinically relevant cell numbers. The first commercial MSC-based products are also addressed, as well as the remaining challenges to the widespread use of MSC-derived products.

## 1. Historical Overview

The first evidence that nonhematopoietic stem cells were present in the bone marrow (BM) and that these cells could be the source of fibroblasts involved in the wound repair process was observed by pathologist Cohnheim in 1867 [1]. However, only a century later (50 years ago), these cells were isolated and cultured *in vitro* [2]. Friedenstein and colleagues found that, when culturing cells from the bone marrow of rats, there was a population of nonhematopoietic cells morphologically similar to fibroblasts that adhered to the plastic of the culture flask. These cells were then referred to as a colony-forming unit fibroblast (CFU-F) and were capable of self-maintenance, differentiation *in vitro* into other cell types (adipocytes, chondrocytes, and osteocytes), and supporting hematopoietic stroma when a single CFU-F was retransplanted *in vivo* [3]. In 1988, Owen proposed the existence of a stromal system, with a stromal stem cell (CFU-F) at the base of hierarchy, popularizing the stromal cell terminology [4]. All these data were generated from animal models. The subsequent studies have failed to identify cells with osteochondrogenic potential in human marrow [5, 6]. Only in 1992, Haynesworth and colleagues enriched and expanded cells in culture with osteochondrogenic potential from human marrow [7].

In the early 90s, the differentiation and *in vitro* proliferation potential was interpreted as indicative of *in vivo* multipotency and self-renewal, characteristics of the “stemness” [8]. Thus, the term mesenchymal stem cell (MSC) was proposed by Caplan for progenitor cells isolated from human adult bone marrow (BM) as an alternative to “stromal” or “osteogenic” stem cell and gained wide popularity [9, 10]. Although BM is still the most common source of MSC, other sources have also been identified such as adipose tissue [11], synovial membrane [12], umbilical vein [13], umbilical cord blood [14], and dental pulp [15], showing features comparable to BM-derived MSC cells.

Ease of isolation and expansion, as well as the *in vitro* multipotentiality, rapidly positioned MSC as a promising therapeutic agent in regenerative medicine and made them the subject of intensive clinical research [8]. The first reports of MSC clinical use occurred between 1995 and 2000 for the treatment of patients with cancer and osteogenesis imperfecta [16–18]. The results of these first clinical studies demonstrated the MSC therapeutic potential as well as the feasibility and safety of such treatments. At that time, it was assumed that MSC could engraft and differentiate into multiple tissues to replace damaged cells [19].

The heterogeneity of MSC isolation, culture methods, and the consequent difficulty to compare the results obtained in clinical and nonclinical studies, conducted between 1990 and 2000, encouraged the International Society of Cellular Therapy (ISCT) to propose criteria for MSC classification in 2006. According to the ISCT definition, “multipotent mesenchymal stromal cells” should be adherent to plastic, positive for CD105, CD73, and CD90 and negative for the expression of CD45, CD34, CD14 or CD11b, CD79 or CD19, and human leukocyte antigen class II, and should also be able to differentiate *in vitro* into osteoblasts, adipocytes, and chondroblasts [20, 21].

**Figure 1:**
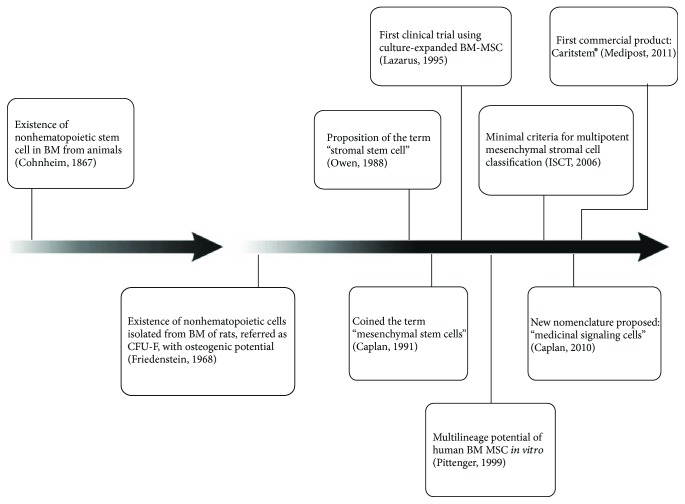
Schematic representation of the main findings related to MSC discovery, characterization, and clinical application throughout the years.

After the first clinical studies, researchers have shown that infused cells survived for short periods in the human body and had limited ability to differentiate *in vivo*. Despite this, the therapeutic effects were still observed even after the “disappearance” of the cells [19]. It was then confirmed that the main therapeutic effect of these cells is related to their immunomodulatory properties based on the capacity of MSC to secrete cytokines and growth factors, acting as multidrug delivery vehicles [22]. As a result, in 2010, Caplan proposed a new nomenclature: “medicinal signaling cells” (MSC) [23].  Figure 1 summarizes the main findings related to MSC discovery, characterization, and clinical applications.

Whether for their regenerative or immunomodulatory potential, MSC have been explored in numerous clinical studies for the treatment of hematological, inflammatory, and autoimmune diseases; graft-versus-host disease; heart, liver, kidney, and lung diseases in the last 15 years. Other properties have brought MSC into the spotlight, including the secretion of soluble active factors, ability to differentiate into several cell lineages, immunomodulatory properties, and migration to the site of injury [24]. Furthermore, MSC can be used for autologous and allogeneic therapies due to the lack of expression of major histocompatibility complex (MHC) class II and the absence of costimulatory molecule expression on their surface [25]. A more in-depth overview of the current clinical status of MSC, mechanisms of action, secretion of active factors, and MSC properties can be found in the works previously described [20, 26–29].

## 2. MSC Manufacturing: From Conventional Cultures to Bioreactors

Despite the vast potential, the MSC therapeutic use is still limited by the need for *in vitro* expansion due to the low frequency of these cells in the tissues of origin (frequency in the bone marrow, e.g., is 0.001–0.01%) [30] and by the high doses required for an infusion (1–100 × 10^6^ cells/kg of patient). As a result, many efforts have been focused on the development of expansion technologies to obtain sufficient numbers of cells with adequate therapeutic quality. Although MSC are often used in an allogeneic scenario, their autologous use can also be employed depending on the therapeutic application. This choice, scale-out versus scale-up, shall have a great impact on the manufacturing process production and, consequently, on the cost of goods. For MSC autologous use, as a lower cell quantity is required, the scale-out approach can be followed, increasing the number of planar culture systems (multiple flasks in cell factories, preferably fully automated). Considering the MSC allogeneic use, it is possible to produce a large number of cells in bioreactor systems (scale-up approach) and to create a robust cell bank to supply cells for all therapies [31].

**Figure 2:**
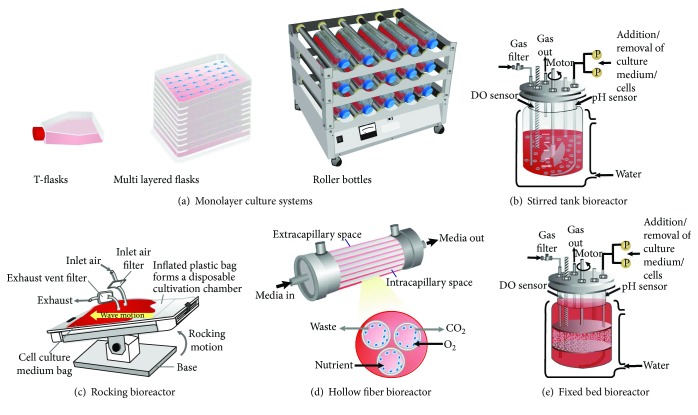
Schematic representation of monolayer culture systems and bioreactors used for MSC expansion.

Monolayer culture or flat two-dimensional flasks are the traditional and widespread technique for MSC expansion due to its simplicity, low cost, and easy handling (Figure 2(a)). It consists of a single compartment where nutrients are diffused to cells and gas exchange (CO_2_ and O_2_) occurs only at the medium/gas interface [32]. Single and, specially, multilayer vessels have been used to progress several cell therapy products into mid-to-late-stage clinical development. The scale-up of this traditional culture process usually involves commercially available multilayered cell factories such as Nunc Cell Factories and Corning Cell Stacks [33]. This culture system is designed to offer a large surface for cell growth by increasing the number of single stack units and has been used by several investigators for MSC expansion [34–38]. The production of 0.45–2.5 × 10^8^ cells can be achieved in 10-layer vessels [39] and has successfully been scaled out to 50–70 vessels (400,000 cm^2^) [40]. Clinically relevant cell numbers can be obtained in 40-layer vessels (~1 × 10^9^cells). However, it is important to emphasize that 40-layer units need an automated cell factory manipulator (ACFM) and a large floor incubator [41].

Despite the effectiveness in promoting MSC expansion, the monolayer culture technology has a number of limitations: excessive manipulation that can interfere in the functional properties of cells due to enzymatic treatments for successive passages and higher contamination risk due to intense manipulation, lack of control of the culture parameters and cell physiology, costly and prolonged culture for the generation of adequate amounts of cells [42]. The static nature of the culture leads to concentration gradients (pH, dissolved oxygen, nutrients, and metabolites) in the culture medium [32] and therefore a heterogeneous environment. A large number of evidence have demonstrated that the 2D system compromises the potency of MSC, while 3D culture could increase the therapeutic potential of MSC by improving the anti-inflammatory and angiogenic properties, stemness, and survival [43, 44]. Additionally, monolayer culture flasks are considered as “open system,” because their subculture (inoculation, medium exchange, and cell harvesting) is carried out in laminar flow cabinets by direct operator manipulation [45]. Although automation and robotics could minimize the disadvantages listed above [32], this technology is not amenable to scale-up when lots higher than 100 billion of cells are required [33].

An alternative to enlarge scale expansion in conventional static monolayer culture flasks could be the use of roller bottles (Figure 2(a)). It consists of multiple cylindrical bottles placed into a rotating apparatus (allocating hundreds of bottles), which minimizes mass transfer limitations [32]. The cells grow forming a monolayer over nearly all the inner surface of the bottle as the culture medium moves continuously. Although it still represents an open system and intensive labor, it offers a greater surface area for growth per vessel and reduces the medium requirement compared with T-flasks [46]. Roller bottles have been used for MSC tissue engineering applications and expansion [39, 47]. Although this system presents advantages over static culture flasks, Tozetti and coworkers were not able to achieve a satisfactory level of expansion by employing roller bottles compared to T-flasks using MSC from an umbilical cord, even by testing several different culture conditions [39].

The high-level production of cells (at least 1 × 10^6^ cells/kg body weight of an adult patient) in accordance with good manufacturing practices (GMP) and quality standards requires a fully closed, controllable, and scalable culture system [46]. Bioreactor-based cell expansion meets these requirements. The bioreactor can be defined as a culture system in which there is a proper monitoring and control of culture variables such as pH, temperature, oxygen, and carbon dioxide concentration for the maintenance of a homogeneous physicochemical environment for the cells, as well as the support for cell adhesion (adherent cells) when needed. Several bioreactor types have been used for MSC expansion, as it can be seen in Figures 2(b)–2(e) and Table 1. Each one has its own specific features (Table 2) that must be evaluated in order to select the best one considering the application. Generally, the bioreactor must be easy to operate; it enables accurate online monitoring and control of culture parameters and achieves high cell densities. It should also allow the easy harvest of viable cells and must be effective in terms of cost and time. Disposable configurations are available, up to 2000 liters, and have been preferred because of the elimination of the cleaning and sterilization steps [46]. Previously sterilized microcarriers have also been commercialized to facilitate cell production and to ensure greater safety. These single-use technologies (SUTs) are widely used and accepted in the cell therapy industry [48]. Given the trend towards personalized cell therapy, the SUTs will be the first choice in a mid/long-term use.

### 2.1. Stirred Tank Bioreactor

The stirred bioreactors, well characterized and widely used for microbial and animal cell cultures, have been used to avoid the limitations of static culture. Spinner flasks and stirred tank bioreactors are the most widely used stirred systems. In this bioreactor, impellers are used to promote mixing, resulting in a homogeneous culture system, which allows monitoring and controlling culture parameters and the constant removal of samples. Among the main advantages, one can find system homogeneity, friendly operation and scaling-up, and operation versatility (batch, fed-batch, and perfusion). A large number of cells can be produced in just one vessel, thereby avoiding vessel-to-vessel variability (as in the case of multiple T-flasks) and minimizing costs related to labor and consumables [65]. The majority of the commercial FDA-approved biopharmaceuticals is produced using this type of bioreactor. The knowledge acquired and the safety record, regarding its use for cell-derived products, facilitated its application for the expansion of MSC and also of other cell types used for cell therapy purposes.

MSC expansion in stirred tank bioreactors, due to its anchorage-dependent nature, requires the use of microcarriers, small beads (100–300 *μ*m diameter) easily maintained in suspension, that provide the surface for cells to attach and grow. Microcarriers present a high surface area-to-volume ratio, 30 cm^2^/cm^3^ medium for Cytodex-3 microcarrier (GE Healthcare) at 10 g/L, for example, whereas T-flasks have a smaller ratio, 3 cm^2^/cm^3^ medium, which allows to achieve much higher cell yields in suspension culture [65] enabling a time- and cost-saving production. These microcarriers are typically spherical beads differing in material, density, diameter, and surface charge.

The selection of an appropriate microcarrier is a critical variable of an expansion bioprocess and must be based on a systematic methodology. The ideal microcarrier not only should be able to support efficient cell attachment and growth but also should be able to allow an easy cell harvesting without losing MSC properties [66]. One interesting approach to select the best microcarrier of a particular process is the use of small-scale systems capable of evaluating the performance of an individual microcarrier and comparing them based on specific culture parameters (cell growth, cumulative population doublings, harvesting efficiency). Ideally, after a stringent screening protocol with at least 3 replicates, a process can be built around that particular microcarrier and can be reproducible in all stages of clinical development [67]. According to Nienow and coworkers, if your study defines the “ideal” microcarrier and provides a reproducible and transferable methodology, there may not be the need to develop an entirely new one when a new donor is needed to be introduced [68]. Recently, Rafiq and coworkers performed an in-depth study comparing 13 commercially available microcarriers for the expansion of human bone marrow-derived mesenchymal stem cells (hBM-MSCs). The results showed that SoloHill Plastic was the optimal microcarrier choice for BM-MSC expansion based on the criteria defined: extent of cell proliferation on the microcarrier, amenability for xeno-free processing, and efficient cell harvesting.

Although with the possibility of cell damage due to shear stress, one of the main disadvantages of expanding cells in microcarriers, in stirred tank bioreactors, is the formation of cell microcarrier agglomerates that prevents the transfer of nutrients to the cells inside the agglomerate and also impairs cell harvest. A neutrally charged microcarrier (a positively charged microcarrier that attracts cells by electrostatic forces) and biodegradable ones (degradable with enzyme digestion) are preferred, once they avoid a high level of agglomeration. Another approach used to minimize cell/microcarrier agglomeration and also to facilitate the MSC scale-up is the use of bead-to-bead transfer process. The bead-to-bead transfer allows a batch feeding of fresh microcarriers (beads) in order to provide an extra surface area for cell growth, and hence, there is no need for subculturing to maximize cell growth. This strategy could potentially reduce the culture handling and culture reagent supplies, minimize the contaminations, and also reduce costs [66, 69].

The harvesting procedure in a microcarrier-based culture is an essential step since the cells will be the final product. Typically, microcarriers colonized with cells are treated with proteolytic enzymes, and the detached cells are then separated from microcarriers by filtering. The proteolytic enzymes cleave the covalent bonds that were formed between the surface layer of the scaffold and integrins on the cell surface. Trypsin, Tryple™, Accutase™, Alfazyme, Collagenase, and TrypZean™ are examples of enzymes that can be used for MSC detachment from the microcarriers. Excluding trypsin, all the other enzymes mentioned above were favored considering nonanimal origin being fully compliant with GMP standards. There is no consensus regarding the most suitable cell harvesting processing, and according to Salzig and coworkers, the process of detachment yield is influenced by multiple variables that include enzyme type and incubation parameters (concentration, temperature, and duration), static versus dynamic systems (shear stress of stirred systems decreases the cell viability after detachment), and downstream process after cell recovery (additional steps also decrease cell viability) [70]. Nienow and coworkers performed a new cell harvesting method based on theoretical concepts. They proposed a short period (7 min) of intense agitation in the presence of a suitable enzyme (trypsin). By using this protocol, the harvesting efficiency was >95% and cells after harvesting showed all the attributes expected for MSC cells. In addition, the authors suggested that the overall protocol is flexible and could be used for different cell lines and microcarriers by just fine-tuning the enzyme concentration and agitation/time [71]. However, it is important to mention that the cell harvesting procedure is not trivial and it becomes more complex, when the expansion scale increases. Indeed, the majority of published articles have not mentioned harvesting efficiency (%).

The scale-up of human MSC in a 5 L stirred tank bioreactor was described by Rafiq and coworkers, using 2.5 L working volume and a nonporous Plastic P-102L microcarrier. Over a 12-day culture period, the researchers achieved a maximum cell density of 1.7 × 10^5^ cells/mL (6-fold expansion), an amount equivalent to the one achievable from 65 fully confluent T-175 flasks [52]. Other reports in the literature have described the successful expansion of MSC in stirred tank bioreactors using microcarriers [39, 50, 51, 53–57] (Table 1).

The majority of MSC cultures are typically expanded using fetal bovine serum (FBS) as a supplement for culture medium. However, to avoid the risk of transmitting xenogeneic infectious agents and immunization, the scientific community has proposed FBS alternatives such as human serum, platelet-rich plasma, and platelet lysate (hPL) [72]. Ideally, a suitable FBS substitute should present defined composition, reduced risk of contamination, low costs, easy availability, and extended shelf life [73]. Some studies have reported the use of a stirred tank bioreactor for MSC expansion under xenogeneic- (xeno-) free conditions with the use of human serum [39] and chemically defined culture medium [74, 75]. The first FDA approved commercial xeno-free culture medium was StemPro MSC SFM (Invitrogen). Dos Santos and coworkers showed an efficient growth of MSC from adipose tissue and bone marrow cultured in plastic microcarrier with StemPro MSC SFM [53]. Growth on collagen microcarriers at serum-free conditions using StemPro MSC SFM allowed the production of 1 × 10^8^ MSC in 5 days of culture [56]. A more concise review of FBS substitutes can be found in previous reports [46, 69, 73, 76].

Upon the increasing importance of stem cell bioprocessing, the interest in using disposable and single-use technologies has appeared and new approaches have emerged. Then, researchers showed the utility of a single-use 3 L stirred tank bioreactor in combination with collagen-coated microcarriers for human bone marrow-derived MSC (BM-MSC) expansion. MSC propagated in the single-use 3 L bioreactor (Mobius, EMD Millipore) for five days with a 5.2-fold increase in total cell number, from 30 to 150 million cells [49]. Similarly, BM-MSC were propagated in a disposable stirred tank bioreactor (Mobius CellReady 3 L bioreactor, Millipore) achieving viable cell densities of 2.5–2.7 × 10^5^ cells/mL during 12–14 days of culture [51, 77].

### 2.2. Rocking Bioreactor

A rocking (wave) bioreactor is a reliable and attractive option for mammalian cell cultures when good manufacturing practice (GMP) bioprocesses are required. This bioreactor consists of a disposable plastic bag placed on a platform whose agitated fluid motion induces the formation of waves which, in turn, provide good nutrient distribution and excellent oxygen transfer with moderate shear stress, resulting in an optimal culture environment for cell growth. It also presents a minimum risk of contamination (closed system), scalability (up to 500 L), and flexibility. In this culture system, microcarriers are also needed for MSC expansion. Although these features provide a great advantage, compared to other bioreactors, there is only one scientific publication demonstrating its use for MSC expansion, for the best of our knowledge. Timmins and colleagues isolated MSC from a human placenta and expanded in a Xuri bioreactor (GE Healthcare) on two types of microcarriers. After seven days of culture, 10-fold expansion was obtained on the Cytodex-3 microcarrier and 15-fold in the Cultispher-S. According to the authors' estimates and the cell isolation method proposed, 500 grams of a placenta is enough to produce cells for two patients of 70 kg at a dose of 5 × 10^6^ cells/kg after the first passage [62].

### 2.3. Hollow Fiber Bioreactor

Hollow fiber bioreactors are considered a good option for the expansion of MSC due to their relatively homogeneous cultural environment, low shear stress, and fibers for cell adherence. Hollow fiber bioreactors basically consist of porous capillaries (hollow fibers) contained in a parallel outer cylinder. Typically, the cells are inoculated within the fiber (intracapillary space (ICS)). The extracapillary space (ECS), between the cylinder and the fibers, is where the culture medium flows and nutrients diffuse through the pores of the fibers to the ICS, allowing the nutrition of the cells retained therein. Also, metabolic waste produced by the cells can permeate through the fiber and it can be carried by the flow. Recent studies have shown the expansion of MSC from different sources using the commercially available disposable Hollow fiber bioreactor (Quantum® Cell Expansion System, Terumo BCT). Starting with 21 × 10^6^ cells, the authors reported a fold increase (average) of about 10 during 7–17 days, using culture medium supplemented with fetal bovine serum (FBS) [59–61]. Another approach described in the literature relates the use of the Quantum system for the enrichment of MSC from unprocessed bone marrow. A range of 2–58 × 10^6^ MSC cells was obtained from 8 to 32 mL of primary bone marrow aspirates in a period of 15 to 27 days. The cultivation of MSC at the second passage for 13 days led to further 10–20-fold enrichment [58]. This bioreactor is also being used for ex vivo expansion of MultiStem® (adherent stem cell product), which is in clinical trial testing for several diseases like inflammatory bowel disease, graft-versus-host disease, stroke, and acute myocardial infarction. Recently, our group has reported the successful expansion of AT-MSC in the Quantum Cell Expansion System under xenogeneic- (xeno-) free conditions, enabling the generation of clinically meaningful cell numbers (11-fold increase) in a reduced period of time (5 days) [78]. The results obtained a point to a successful cell expansion, encouraging other investigators to use this disposable closed system to expand cells for cell therapy purposes [79].

### 2.4. Fixed-Bed Bioreactor

A fixed-bed bioreactor consists of a column (bed), which contains/holds an immobilized scaffold, where the cells are inoculated. The scaffold must have a high surface area for cell growth and chemical stability. Once the cells remain immobilized on the carrier surface, this system has an advantage of presenting a low shear stress environment. Although this bioreactor allows a three-dimensional cell growth and better mimicking *in vivo* conditions, spatial concentration gradients may occur [32]. A fixed-bed bioreactor using nonporous borosilicate glass spheres as carriers was used for the expansion of the cell line hMSC-TERT. In this work, they used bed volume up to 300 mL and described automated inoculation, cultivation, and harvesting of the cells. Additionally, a model describing the process was developed, based on the collected data, in order to perform calculations for scaling up [80].

The FibraStage bioreactor is a disposable fixed-bed culture system with polystyrene disks (Fibra-Cel® disks) as a scaffold. Our research group tested this culture system for human MSC expansion. After 7 days of culture, it was possible to produce 4.2 (±0.8) × 10^8^ cells, which represents a fold increase of 7.0. This amount of expanded cells is sufficient to infuse six patients (70 kg), considering the number of 1 × 10^6^ cells/kg per patient; therefore, to produce the same amount of cells, it will be necessary to use 120 75 cm^2^ culture flasks. It is worth mentioning however that a low harvesting efficiency in the fixed-bed bioreactor (18% (±0.8)) was attained, due to the insufficient time of enzyme treatment and gentle platform motion, which prevents efficient enzyme diffusion throughout the bed [63]. In another study, Tsai and coworkers demonstrated the feasibility of MSC expansion in a 2.5 L stirred tank packed with the same scaffold used in our study. After 9 days of expansion, a 9.2-fold increase in the cell number was achieved. However, the authors did not mention harvesting efficiency (%) [64]. The company Pluristem Therapeutics (based in Israel) is expanding placental-derived mesenchymal cells (PLX) using a proprietary fixed-bed bioreactor (PluriX 3-D bioreactor) in combination with Fibra-Cel disks [81].

## 3. MSC Downstream Processing

MSC downstream processing (DSP) involves several complex steps, after cell detachment from the scaffold, which include microcarrier (scaffold) removal (clarification), volume reduction for concentration, cell washing followed by formulation, and cryopreservation. Few studies have described the MSC downstream process due to a limited number of DSP technologies available to fulfill the allogeneic cell therapy scenario. In order to obtain a highly pure cell product with adequate viability and functionality, the whole DSP process must meet specific requirements, including reduced processing time, high volume reduction, efficient washing (to diminish the impurity levels to <1 ppm), low shear stress conditions and additionally, the system needs to be closed, automated and scalable under GMP standards [82].

Aiming an efficient GMP-grade downstream process, Cunha and coworkers evaluated for the first time the use of dead-end filtration and tangential flow filtration (TFF) for the clarification and concentration of MSC, respectively. The results showed that polypropylene filters with pore sizes higher than 75 *μ*m could provide an efficient microcarrier removal and polysulfone membranes with pore sizes higher than 0.45 *μ*m (hollow fiber cartridge) were able to concentrate the cells to a factor of ten (viability > 80%) [83]. One year later, the same research group performed another study to improve the established TFF-based strategy. Using negative mode expanded bed adsorption (EBA) chromatography with a new multimodal prototype matrix based on core-shell bead technology, they were able to improve the washing step by more than 10-fold recovering 70% of viable and functional MSC. Moreover, the chromatographic step enables a single-pass operation decreasing the time of cell handling [84]. Alternatively, to the use of TFF, single-use recovery equipment such as closed continuous fluidized bed centrifuges (kSep® Systems) has also been explored [85]. These systems have the volume capacity ranging from 400 mL to 6 L and operate via counter-flow centrifugation allowing volume reduction and washing in a low-shear stress environment [82].

After the concentration and washing steps, the cells are then formulated using a specific cryopreservation buffer. Given that MSC-based therapy is intended for allogeneic use, a large “off-the-shelf” inventory must necessarily be created (many doses per lot). Then, vial filling at a large scale has to be performed using automated systems (such as Crystal® Px) and controlled-rate freezers will also be required to process thousands of vials per batch. The combination of all these automated and closed systems will enable the maintenance of cell product quality [82, 86].

## 4. MSC Quality Control

MSC-based therapies are considered advanced therapy medicinal products (ATMPs) and must be manufactured according to good manufacturing practices (GMP) (manufacturing authorization is required) [10]. There is no consensus regarding quality control standards among countries, and each research center should discuss the application on a case-by-case basis with their local regulatory agency authority. Commonly, assays to assess the quality safety and efficacy of MSC are performed during their production for the final clinical use. It includes cell identity morphology growth characteristics sterility karyotype and efficacy tests.

In order to assess the MSC identity, researchers should follow the three minimal criteria proposed by the ISCT: adherence to the plastic, expression of a specific surface antigen, and trilineage differentiation, as already mentioned [21]. The MSC phenotypic profile is considered a release criterion, and for this reason, controls should be performed to guarantee the validity of results [87]. Regarding morphology, MSC should maintain a spindle-shaped morphology throughout the culture. A drastic morphology change could affect MSC response and commitment [88]. Similarly, cellular growth should be monitored at each passage and expressed in terms of population doublings (PD). The number of population doublings (PD) could be calculated using the equation PD = log(FI)/log(2), where FI is the cell culture fold increase estimated by the number of final cells/number of cells inoculated. Viability should be maintained at >90% and could be assessed using the trypan blue exclusion method or by using propidium iodide (flow cytometry). Ideally, the cell expansion should not exceed 20 population doublings to avoid the senescence process [10].

Once MSC need to be expanded *in vitro* for an extended period of time, the maintenance of genomic stability has to be assured by performing karyotype analysis, comparative genomic hybridization (CGH) array, or fluorescence in situ hybridization (FISH) [89]. In 2013, an expert group, including people from European Regulatory Authorities, reached an agreement on several issues and proposed a statement: culture conditions should be carefully chosen to avoid a high proliferative rate, and the number of population doublings should be kept to a minimum, avoiding chromosomal abnormalities. Conventional karyotyping has to be performed to evaluate putative chromosomal aberrations [90].

The assessment of contamination risk that could potentially affect the efficacy, safety, and quality of MSC has also to be considered. Contamination by bacteria, fungi, mycoplasma, and bacterial endotoxin should be documented and evaluated. These tests should be performed not only in the final cellular product but also in the entire manufacturing process, including reagents and starting materials. In 2014, Gálvez and coworkers described an efficient quality control program (QCP) according to the European Pharmacopoeia to detect contamination during the manufacturing of autologous hMSC for clinical application. All the methods, procedures, and validations can be accessed in the article published [91].

Efficacy could be assessed using *in vitro* and *in vivo* (animals) experiments during the preclinical phase of process development. As the efficacy tests differ among the intended clinical applications, no concluding recommendation and no specific tests are required by the authorities as a release criterion. It is well recognized that MSC possess immunosuppressive potential, showing the best clinical results so far in immunological-based diseases [87]. Therefore, the immunosuppressive capacity of MSC could be tested *in vitro* by different immunological assays, such as the inhibition of T-lymphocyte proliferation and cytokine release assay. Although these tests are not mandatory, they may represent a fundamental step towards MSC characterization and future clinical application, being in accordance with GMP requirements.

The use of process analytical technologies (PAT), under the QbD (Quality by Design) umbrella, for regulating product quality must also be considered. The PAT system, developed by FDA (Food and Drug Administration), considers science and engineering principles for assessing and mitigating risks related to poor product and process quality. The quality, therefore, has to be done as an in-process online control rather than only final testing [92]. PAT principles, as well as QbD, are increasingly being incorporated into the bioprocessing industry. In the cell therapy field, however, the application of PAT concepts is challenging because of the difficulty of fully characterizing a living cell and obtaining relevant data in real time [93]. For further reading, see [94–96].

## 5. Commercialization of MSC-Based Products

The use of MSC as a therapeutic product has been extensively explored in the context of clinical studies (203 MSC-based clinical trials, either ongoing or completed, are found on http://clinicaltrials.gov). In general, these studies have concluded that their use is safe, feasible, and effective in certain cases and conditions. However, only a few commercial products have been approved by regulatory agencies. The first commercial (allogeneic) product, Caritstem®, based on MSC derived from umbilical cord blood and produced by Medipost, was only approved for the treatment of traumatic and degenerative osteoarthritis in 2011 in Korea. FCB-Pharmicell (Korea) obtained the approval of the second commercial (autologous) product, HeartiCellgram® (based on MSC derived from the patient's own bone marrow), indicated for the treatment of acute myocardial infarction. The company produces 50–90 million cells per patient, and this product is infused into the coronary arteries [97].

Canada was the second country to approve an allogeneic product based on MSC (bone marrow of healthy volunteers), Prochymal® (Remestemcel-L), for the treatment of children with graft-versus-host disease in 2012 (Osiris Therapeutics Inc.). Other Osiris' product line available in the market also includes Cartiform® for cartilage repair, Grafix® for acute and chronic wounds, and Stravix® for wound repair [98]. Another product approved for marketing by South Korea's Food and Drug Administration in 2012 was Cupistem® (Anterogen). It consists an autologous adipose-derived mesenchymal cell treatment to reduce inflammation and regenerate damaged joint tissues, indicated for the treatment of Crohn's fistula.

More recently, Mesoblast has launched in the market TEMCELL® product, an allogeneic mesenchymal stem cell product indicated for the treatment of acute radiation injury, Crohn's disease, graft-versus-host disease, type I diabetes, and myocardial infarction. The product was fully approved in Japan (Japanese Ministry of Health, Labour and Welfare) in 2015 and afterwards approved in New Zealand and Canada [99].

## 6. Remaining Challenges

The transition from monolayer-based expansion to bioprocess using bioreactors, already experienced by the pharmaceutical industry in the production of viral vaccines and recombinant proteins, enabled not only the increase in the number of cells produced and the reduction of process costs but also the constant monitoring and control of important cell growth parameters, improving the quality and safety of the cells produced in accordance with good manufacturing practices. Notwithstanding the vast knowledge related to cell culture in bioreactors acquired by academia and industry for the production of cell-derived products, its application in the production of cell-based products had to consider the peculiarities of this new type of product, mainly referring to post-expansion cellular safety and functionality. Cell-based formulations, for example, cannot undergo viral inactivation processes along the purification as the recombinant proteins, so the production process must be conducted in a manner to ensure the complete absence of contaminations. Due to their primary nature, MSC cannot be cultivated indefinitely, due to their senescence and eventual loss of important functional properties. The use of MSC with less than 20 population doublings has been suggested for clinical applications to ensure safety and efficacy [65]. It is important to keep in mind that a “one-size-fits-all” bioprocess platform is unlikely, due to the heterogeneity of cell types, protocols, reagents, and disease indications. A large-scale manufacturing protocol has to be tailored for each specific clinical application. It must also consider the impact of donor age on cell proliferation and biological properties. Choudhery and coworkers showed that aged adipose tissue-derived MSC (>60 years) displayed senescent features when compared with young donor cells (<30 years), as well as reduced viability, proliferation, and differentiation potential. The presence of age-related diseases, such as diabetes and heart failure, can also negatively affect cell functionality [100]. According to Petry and colleagues, the gender of the cell donor had no influence on cell growth and metabolism [101].

The majority of works describing the large expansion of MSC employs the use of fetal bovine serum (FBS) for product manufacture, and a strategy to integrate xeno-free culture needs to be addressed. However, it is worth noting that unlike the recombinant protein production process, regulatory agencies still allow cell expansion in FBS-containing media, although they recommend withdrawing this component and other animal components from the production bioprocess [26]. Currently, as previously mentioned, some groups are testing new xeno-free culture medium formulations (hPL, human serum, chemically defined media, etc), overcoming ethical issues related to FBS usage and improving the expansion of MSC for clinical applications in a safe and reproducible manner.

One challenging requirement for MSC production and therapeutic use is to establish minimum standards for quality control. Ideally, the cell manufacturing bioprocess should be reproducible and fast and the released product should be systematically tested for identity, safety, purity, and efficacy as already mentioned. Regardless of the constant efforts from scientific community and industries, there is still no consensus on quality control assays for the production of MSC for therapeutic purposes.

Another remaining challenge is related to the complexity of cell-based product production process and, consequently, the high cost of goods (COG). Prices from approximately $25,000/dose up to $40,000/dose have been reported. As a result, the search for economically viable production processes will be critical if cell therapy products are intended to achieve the commercial manufacturing success of biopharmaceuticals. This issue may be related to the fact that despite the high therapeutic potential and the numerous ongoing clinical studies, few MSC-based products have been approved in the market.

Even with all these remaining challenges, progresses in cell manufacturing with the use of bioreactors and improvements in cell characterization and quality control will certainly accelerate the therapeutic use of MSC to treat several incurable diseases.

## Figures and Tables

**Table 1:** MSC cell expansion in monolayer culture systems and bioreactors.

Type	Culture medium	Working volume (mL)	MSC source/culture conditions	Time in culture (days)	Fold increase	Final cell number	Reference
10-layer HYPER flasks (Corning)	*α*-MEM + 15% AB human serum	560	UCM MSC	11	13	45 × 10^6^ cells	[39]
Roller bottles (Greiner)	*α*-MEM + 15% AB human serum	200	UCM MSC	6	7.0	29 × 10^6^ cells	[39]
3 L stirred tank bioreactor (EMD Millipore, Mobius)	DMEM + 10% FBS	2000	BM and AT MSC on collagen-coated microcarriers	5	5.2	n.r.	[49]
1 L bioreactor (Sartorius Biostat B-DCU)	DMEM + 10% FBS	1000	BM MSC cultivated on Cytodex-3 microcarrier	8	12	600 × 10^6^ cells	[50]
3 L stirred tank bioreactor (Merck-Millipore, Mobius™ CellReady 3L)	DMEM + 10% FBS	2400	BM MSC cultured on collagen-coated SoloHill microcarriers	12	62	270 × 10^6^ cells/L	[51]
5 L stirred tank bioreactor (Sartorius, Biostat B Plus)	DMEM + 10% FBS	2500	BM MSC cultivated on Plastic P-102L microcarrier	12	>6	420 × 10^6^ cells	[52]
1.3 L stirred tank bioreactor (New Brunswick, Bioflo)	StemPro® MSC SFM XenoFree	800	BM and AT MSC cultivated on nonporous plastic microcarrier	7	n.r.	110 × 10^6^ cells for BM MSC and 45 × 10^6^ cells for AT MSC	[53]
2 L stirred tank bioreactor (UniVessel SU, Sartorius)	Serum-reduced (5%) medium	2000	AT MSC cultivated on Pronectin F microcarrier	7	27	540 × 10^6^ cells	[54]
2 L stirred tank bioreactor (Biostat B-DCU, Sartorius)	*α*-MEM + 10% FBS	800	Fetal BM MSC cultivated on Cytodex-3	7	16	0.46 × 10^5^ cells/cm^2^	[55]
2.5 L stirred tank bioreactor (New Brunswich, Celligen 310)	StemPro MSC SFM XenoFree	800	UCM MSC cultivated in Cultispher-S microcarrier	5	5.3	110 × 10^6^ cells	[56]
2.5 L stirred tank bioreactor (New Brunswich, Celligen 310)	*α*-MEM + 15% AB human serum	800	UCM MSC cultivated on Plastic P-102L microcarrier	7	8.9	79 × 10^6^ cells	[39]
2 L stirred tank bioreactor (Sartorius, UniVessel® SU bioreactor)	Mesencult™-XF	1000	BM and AT MSC cultivated on Synthemax® II microcarrier	7	14 for BM MSC and 16 for AT MSC	680 × 10^6^ cells for BM MSC and 820 × 10^6^ cells for AT MSC	[57]
Hollow fiber (Quantum, Terumo BCT)	DMEM + 10% Human Platelet Lysate	n.r.	BM MSC	13	10–20	2–58 × 10^6^ cells	[58]
Hollow fiber (Quantum, Terumo BCT)	DMEM + 10% FBS	n.r.	BM MSC	7	5.5–14	110–276 × 10^6^ cells	[59]
Hollow fiber (Quantum, Terumo BCT)	*α*-MEM + 10% FBS	n.r.	AT MSC	17	4.7	99 × 10^6^ cells	[60]
Hollow fiber (Quantum, Terumo BCT)	DMEM + 10% FBS	n.r.	PD MSC	8	16–22	316–444 × 10^6^ cells	[61]
Rocking bioreactor (GE Healthcare)	LGDMEM + 20% FBS	2000	Pl MSC cultivated on Cytodex-3 and Cultispher-S microcarrier	7	10 for Cytodex-3; 15 for Cultispher-S	n.r.	[62]
Fixed-bed bioreactor (New Brunswich)	*α*-MEM + 10% FBS	500	UCM MSC in a Fibrastage® bioreactor with FibraCel disks	7	7	420 × 10^6^ cells	[63]
Fixed-bed bioreactor (New Brunswich, Celligen 310)	*α*-MEM + 10% FBS	2500	BM MSC	9	9.2	10–92 × 10^6^ cells	[64]

n.r.: not reported; FBS: fetal bovine serum; UCM MSC: mesenchymal stem cell derived from umbilical cord; BM MSC: mesenchymal stem cell derived from bone marrow; AT MSC: mesenchymal stem cell derived from adipose tissue; PD MSC: mesenchymal stem cell derived from periosteum; Pl MSC: mesenchymal stem cell derived from placenta.

**Table 2:** Main features of the culture systems and bioreactors employed for MSC manufacturing.

Features	Multilayered flasks	Stirred tank bioreactor	Rocking bioreactor	Fixed-bed bioreactor	Hollow fiber bioreactor
Homogeneity	No	Yes	Yes	No	Moderate (spatial concentration gradients)
Culture parameter control and monitoring	No	Yes	Yes	Yes	Yes
Scale-up	Limited	Moderate	Moderate (up to 500 L)	Moderate	Moderate
Contamination risk	High (open system)	Low (closed system)	Low (closed system)	Low (closed system)	Low (closed system)
Shear stress	No	High	Moderate	Low	Low
Oxygen transfer	Low	High	High	Moderate	High
Culture operation mode	Batch	Batch, fed-batch, perfusion	Batch, fed-batch, perfusion	Batch, fed-batch, perfusion	Perfusion
Cell harvesting	Easy	Difficult	Difficult	Difficult	Easy
